# Central Nervous System Involvement in Henoch-Schonlein Purpura in Children and Adolescents

**DOI:** 10.1155/2017/5483543

**Published:** 2017-01-21

**Authors:** Iliyana H. Pacheva, Ivan S. Ivanov, Krastina Stefanova, Elena Chepisheva, Lyubov Chochkova, Dafina Grozeva, Angelina Stoyanova, Stojan Milenkov, Penka Stefanova, Anna Petrova

**Affiliations:** ^1^Department of Pediatrics and Medical Genetics, Medical University-Plovdiv, Plovdiv, Bulgaria; ^2^Department of Pediatric Surgery, “St. George” University Hospital, Plovdiv, Bulgaria; ^3^Department of Imaging Diagnosis, “St. George” University Hospital, Medical University-Plovdiv, Plovdiv, Bulgaria

## Abstract

Central nervous system (CNS) involvement in Henoch-Schonlein purpura (HSP) is rare but poses diagnostic difficulties. The aim of the study was to establish the frequency of CNS involvement in HSP, to analyze its clinical characteristics and do a literature review. Medical files of patients with HSP admitted at the Department of Pediatrics, Plovdiv, were studied retrospectively for a five-year period (2009–2013). Diagnosis was based on the American College of Rheumatology criteria. Out of 112 children with HSP 1 case (0.9%) had CNS involvement presenting as Posterior Reversible Encephalopathy Syndrome (PRES), which may be a result of CNS vasculitis or arterial hypertension. It was an 8-year-old girl with atypical HSP which started with abdominal pain requiring surgery. On the third day after the operation a transient macular rash and arterial hypertension appeared, followed by visual disturbances, hemiconvulsive epileptic seizures, postictal hemiparesis, and confusion. Head CT showed occipital hypodense lesions and MRT-T2 hyperintense lesion in the left occipital lobe. The patient experienced a second similar episode after 2 weeks when palpable purpura had also appeared. Neurological symptoms and MRI resolved completely. HSP can be an etiological factor for PRES in childhood. Although PRES is a rare complication of HSP, clinicians must be aware of it and avoid diagnostic and therapeutic delays.

## 1. Introduction

Henoch-Schonlein purpura (HSP) is a systemic vasculitis involving the small vessels. It occurs mainly in children; over 75% of patients are under 10 years of age [1]. Its incidence is 10–20 per 100,000 children [2–5]. Most commonly affected are the skin, joints, gastrointestinal tract, and kidneys. CNS involvement in HSP is rare (0.65–8%) but poses diagnostic difficulties and sometimes has long-term neurological sequelae [3, 6, 7]. Neurologic manifestations of HSP were first described by Osler [8] in 1914 as transitory hemiparesis and decreased level of consciousness as a result from either oedema or brain hemorrhage. There are few case reports about the neurological manifestations of HSP (headache, seizures, hemiparesis, aphasia, cortical blindness, and impaired consciousness) [9–18] and even fewer studies [19, 20]. Many of them are written in the past century, when MRI with new sequences was not readily available. Moreover histologic confirmation of CNS involvement in HSP occurred casuistically [21, 22]. That is why the pathogenic mechanisms of CNS involvement in reported cases remain unclear. Contemporary studies on the characteristics of CNS involvement in HSP with the use of modern imaging methods are needed to extend our knowledge of this category.

## 2. Aim

The aim is to establish the frequency of CNS involvement in children with HSP and to analyze its clinical characteristics and do a literature review.

## 3. Patients and Methods

A retrospective study of the medical files of patients up to 18 years of age, admitted to the Pediatric Department of “St. George” University Hospital, Plovdiv, with a diagnosis of HSP during a five-year period (2009–2013), was performed. Diagnosis was based on the American College of Rheumatology criteria [23]. Patients were diagnosed with HSP if at least two of the criteria were met: (1) palpable purpura, not related to thrombocytopenia; (2) age <20 years at disease onset; (3) bowel angina; (4) histologic changes showing granulocytes and the walls of arterioles and venules. Other types of vasculitis and hemorrhagic diathesis were excluded by detailed medical history, physical and neurological examination, additional functional, imaging, and laboratory investigations. All medical files were screened for neurological manifestations.

The work has been carried out in accordance with the Code of Ethics of the World Medical Association (Declaration of Helsinki) for experiments involving human subjects.

## 4. Results

**Figure 1:**
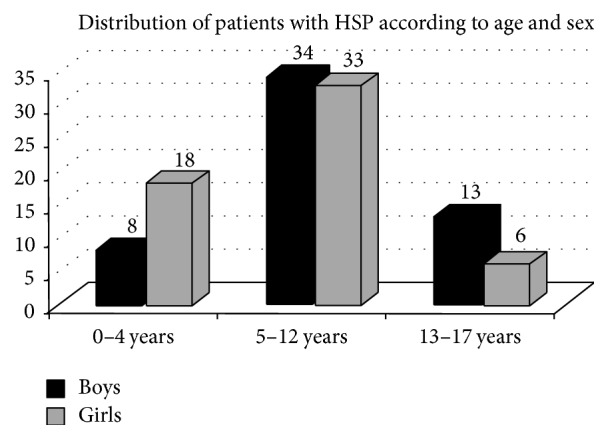
Distribution of patients by age and sex.

The age and sex distribution of patients are presented in Figure 1.

Skin rash was the only clinical feature of the disease in 12 children (11%). Sixty-eight patients (61%) had joint manifestations, arthralgia or arthritis, while 52 of them (46%) showed signs of gastrointestinal tract involvement. There were renal manifestations in 34 children (30%); the majority of them had mild proteinuria (<20 mg/m^2^/h) and hematuria; some had either hematuria or proteinuria. One child developed acute glomerulonephritis complicated with acute renal failure and later bilateral ureteral stenosis. Six children showed signs of mixed nephritic-nephrotic syndrome and had arterial hypertension, but not severe.

Out of 112 children with HSP there is only one case (0.9%) with CNS involvement which presented as Posterior Reversible Encephalopathy Syndrome (PRES). It is probably due to CNS vasculitis but could also be associated with transitory, short-lived arterial hypertension.

## 5. Case Presentation

The case with CNS involvement (a previously healthy 8-year-old girl) had an atypical clinical presentation with many diagnostic challenges: it started with colicky abdominal pain, with signs of acute abdomen, necessitating surgical treatment. Primary aseptic peritonitis was established. On the third day after the operation a transient macular rash appeared, followed by neurological symptoms, visual disturbance, right-sided hemiconvulsive epileptic seizures, postictal right-sided hemiparesis, and confusion, lasting around 30 min. There was transient arterial hypertension (BP 150/100 mmHg) after the application of methylprednisolone and midazolam. Her consciousness level returned to normal within a few hours and she had no residual neurological symptoms and signs. Some short-lived rises in her blood pressure were detected; BP reached 150/105–145/110 mmHg without other symptoms.

Ten days after the first operation the girl presented again with severe abdominal pain and subileus, requiring second laparotomy. Diarrhea with blood and mucous occurred 2 days later. She developed transitory neurological symptoms (acute headache, followed by reduced consciousness to stupor and three secondary generalized or complex partial seizures) again 14 days after the initial presentation of neurological disturbances. Raised blood pressure up to 160/110 mmHg was again detected which returned to normal within the next few hours. Then scarce palpable purpura around her ankles and proximal parts of her arms was noted which disappeared within couple of days. She had no further abdominal or neurological symptoms and recovered completely.

### 5.1. Laboratory, Functional, and Imaging Investigations

FBC showed leukocytosis up to 25 × 10^9^/l with neutrophilia and ESR 30 mm per hour.

CRP was mildly elevated to 40 mg/l.

Biochemistry (electrolytes, urea, creatinine, protein, albumin, transaminases, amylase, fibrinogen, and lactate); coagulation studies, including D-dimers; immunological studies (IgA, IgM, IgG, С3, and С4) were within reference range. Anti-dsDNA, anti-SS-A, anti-SS-B, anti-Sm, anti-Sm/RNP/, ANCA-MPO, anti-cardiolipin antibodies were negative.

Blood culture and stool culture were negative. Arterial blood gases and urinalysis were normal.

Occult blood in stools was positive.

### 5.2. Histology

Appendix was with hyperplastic lymphoid follicles and hemorrhagic-serous exudate on the serosa.

**Figure 2:**
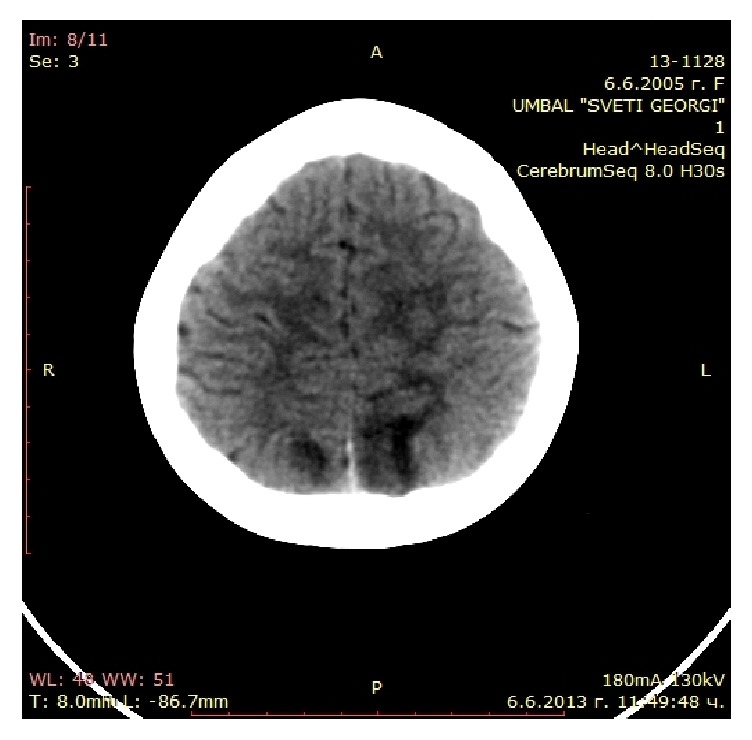
Head CT after the first episode with neurological symptoms: subcortical parietooccipital, parasagittal hypodense lesions in the white matter bilaterally but more on the left.

Head CT after the first episode with neurological symptoms showed subcortical parietooccipital, parasagittal hypodense lesions bilaterally but more on the left. The lesions did not change after the application of contrast agent (Figure 2). The CT findings remained the same after the second episode of neurological symptoms.

**Figure 3:**
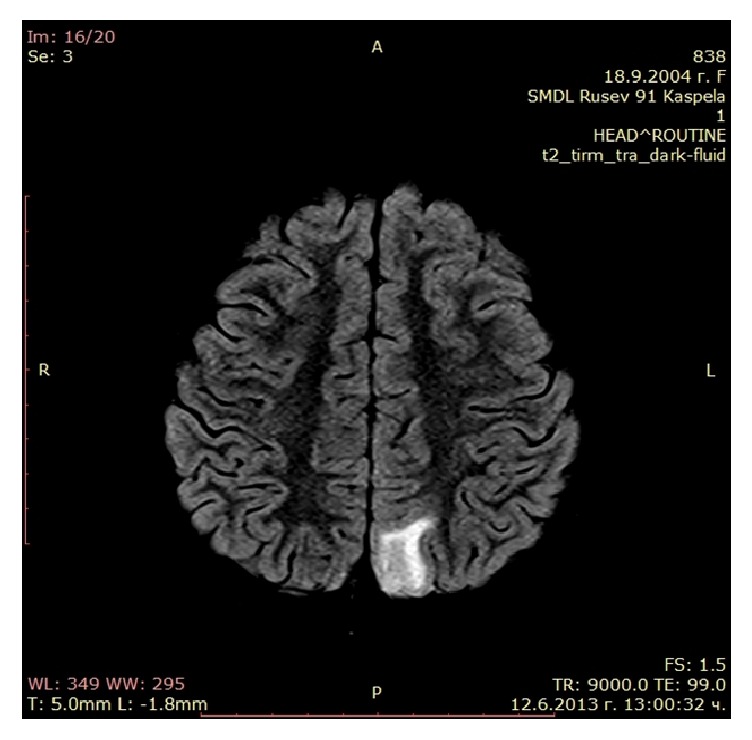
Cerebral МRI: hyperintense in Т2 and FLAIR lesion in the left occipital area.

MRI 6 days after the first episode with neurological symptoms showed hypointense on T1, hyperintense on T2, and FLAIR lesion in the left occipital area subcortically without contrast enhancement (Figure 3). MR angiography was normal.

### 5.3. EEG

EEG revealed slow-wave activity in the left parietooccipital region after the first episode with neurological symptoms and slow-wave activity in the right occipital region after the second episode.

The final diagnosis was Henoch-Schonlein purpura with involvement of skin, gastrointestinal system, and CNS as PRES after excluding other systemic vasculitides, primary CNS vasculitis, PRES in arterial hypertension of other origin, and disturbances of the homeostasis, Crohn's disease, primary pneumococcal peritonitis with sepsis, thrombophilia, and MELAS.

Treatment included antibiotics, rehydration therapy, fresh frozen plasma, methylprednisolone, phenobarbital, midazolam, and enalapril (as antihypertensive).

The slow-wave activity on EEG had disappeared at 1-month follow-up. No abnormalities were detected by brain MRI after 4 months, which supported the diagnosis of PRES.

## 6. Discussion

Our study confirms the data of other authors that CNS involvement in HSP is very rare (less than 1%) [6, 24]. It occurs mainly in patients with arterial hypertension or atypical presentation, as in our case [5, 19]. The reported case had an unusual clinical course; it started with abdominal symptoms severe enough to require surgery twice; the rash appeared late and was scarce; CNS was involved as PRES.

There are large series of patients with no neurological symptoms [4, 25]. In contrast to them one study by Ostergaard and Storm [20] points out that 28% of patients with HSP had headache without other neurological symptoms. In the same study EEG abnormalities in the form of focal or diffuse slow-wave activity and paroxysms are described in 55% of the patents [20]. Headache is reported in the literature as the most frequent neurological symptom in HSP, but in lower percentage of 3–9% [7, 9]. Its pathogenic mechanism is not thoroughly understood and it does not mean obligatory CNS involvement. It could be the result of either arterial hypertension without hypertensive encephalopathy or febrile illness which provoked HSP. As our study is retrospective and any short-lived headache might not have been documented in the files we cannot make any conclusion about the occurrence of mild transient headache (which with such characteristics probably could not be a result of CNS involvement) among our patients.

The clinical signs and symptoms of CNS dysfunction established by Garzoni et al. [19] are altered consciousness (58%); seizures (14%); focal neurological deficit (26%); visual disturbances (24%); speech disturbances (10%). Patients with headache but without any abnormal neurological signs were not included in their study.

CNS involvement in HSP may be a result of CNS vasculitis or associated with arterial hypertension in HSP nephritis [3, 9, 19].

CNS vasculitis in HSP may present as edema, ischemia, ischemic infarction, and hemorrhage [5, 9, 19]. As histological confirmations are very rare, the diagnosis is usually made by imaging studies [11, 14, 26]. The parietooccipital areas are most commonly affected with edema or ischemic infarcts, rarely hemorrhages [18]. Even rarer presentations of HSP are involvement of the peripheral nervous system as neuropathy or multiple mononeuritis [9, 19, 27].

In our case CNS was involved as PRES (based on clinical and imaging criteria) with only intermittent arterial hypertension and without signs of nephritis as hematuria or proteinuria. That is why we think that the vasculitis was the leading factor in the pathogenesis of PRES. The arterial hypertension, although of an uncertain pathogenic mechanism, probably also had a role as during both episodes with neurological symptoms transitory rises of blood pressure were detected. The patient did not receive any medications which could, according to the literature, provoke PRES.

None of the other patients with mild arterial hypertension in our study had any neurological symptoms.

PRES is a unique presentation of vasogenic cerebral edema, described by Hinchey et al. [28] in 1996. It is a clinicoradiological syndrome which presents with headache, visual disturbances, seizures, altered consciousness, and focal neurological deficit, as in our patient. The reduced level of consciousness may vary from somnolence to coma. Seizures are most commonly secondary generalized. Arterial hypertension is present in 67–80% of patients with PRES [29]. On CT and MRI there is focal cerebral edema, most often symmetrical, mainly of the subcortical white matter in the parietooccipital areas, like the images of our patient [30]. MRI is better at detecting changes in PRES. It shows hyperintense areas in T2 and FLAIR. МR DWI sequence visualizes vasogenic edema by establishing increased diffusion coefficient and differentiates vasogenic edema from ischemic and other lesions. In our case we were unable to perform MRI with DWI sequence and the diagnosis of PRES was based on the clinical course of the disease with full neurological recovery within 1-2 days and the reversible nature of the lesions on the imaging studies.

Conditions in which PRES may develop are presented as follows.

*Conditions with Risk of PRES (Adapted from Bartynski 2008) [30]*

  Arterial hypertension
  Toxemia of pregnancy (preeclampsia/eclampsia)
  Posttransplantation
    Allo-BMT
    SOT
  Immune suppression
    Cyclosporine
    Tacrolimus (FK-506)
  Infection/sepsis/shock
    Systemic inflammatory response syndrome
    Multiorgan dysfunction syndrome
  Autoimmune diseases
    Systemic lupus erythematosus
    Systemic sclerosis (scleroderma)
    Wegener's
    Polyarteritis nodosa
    Henoch-Schonlein purpura
  Status-postcancer chemotherapy
    Combination high-dose chemotherapy
    Reported miscellaneous drugs
      Cytarabine
      Cisplatin
      Gemcitabine
      Tiazofurin
      Bevacizumab (Avastin)
      Kinase inhibitor BAY 34–9006 h
  Miscellaneous reported associations
    Hypomagnesemia
    Hypercalcemia
    Hypocholesterolemia
    Intravenous immunoglobulin
    High-dose intravenous corticosteroid
    Guillain-Barré syndrome
    Ephedra overdose
    Dialysis/erythropoietin
    Triple-H therapy
    Tumor lysis syndrome
    Hydrogen peroxide
    Dimethyl sulfoxide stem cells

PRES often poses diagnostic difficulties [29]. Systemic vasculitides are rare etiological factors for PRES and they are most commonly Systemic lupus erythematosus (SLE) and Polyarteritis nodosa (PAN) [30, 31]. HSP may play an etiological role in PRES either as a systemic vasculitis or as a cause for arterial hypertension in patients with nephritis. There are few cases of PRES in HSP published in the literature [10, 12, 15, 16, 32–34]. Most commonly it is associated with arterial hypertension in developing nephritis. In two of the cases, however, there is no arterial hypertension [10, 32]. Recently published systematic literature review on the occurrence of PRES in HSP found only 17 cases of HSP complicated by PRES [35].

In PRES there is vasogenic edema as a result of loss of cerebral autoregulation, endothelial dysfunction, and disruption of the blood-brain barrier [28, 30]. The vessels of the vertebrobasilar system have weaker adrenergic innervation and rising of the arterial blood pressure may easily disrupt the autoregulation of the pressure in their perfusion zones. This explains why the pathological changes are most commonly located in the posterior part of the brain [28]. In most cases there is hyperperfusion, but some evidence also suggests vasoconstriction with hypoperfusion [29]. In cases without arterial hypertension PRES occurs due to cytotoxic edema. There is a hypothesis that interleukin-6 and the endothelial growth factor also play a role in PRES in HSP, which might lead to new therapeutic approaches [36].

When adequate homeostasis is maintained, the brain changes usually resolve without any neurological sequelae, as in our case.

## 7. Conclusions

CNS involvement occurs in less than 1% of the cases of HSP. Although neurological complications are rare in HSP clinicians must be aware of them in order to avoid diagnostic and therapeutic delays. HSP can be the etiological factor for PRES in childhood, and MR DWI should be the preferred method for diagnosing PRES.
